# Diversity of oligosaccharides in lipooligosaccharides of *Akkermansia muciniphila* and its anti-atherosclerotic activity

**DOI:** 10.1007/s13659-026-00612-4

**Published:** 2026-05-02

**Authors:** Yuting Zhang, Wang Dong, Jinghan Lin, Jingzu Sun, Ruopeng Yin, Xun Lv, Wenzhao Wang, Tao Wang, Hongwei Liu

**Affiliations:** 1 State Key Laboratory of Microbial Diversity and Innovative Utilization, Institute of Microbiology, Chinese Academy of Sciences, Beijing, People’s Republic of China; 2 Medical School, University of Chinese Academy of Sciences, Beijing, People’s Republic of China; 3 The Laboratory of Microbiome and Microecological Technology, Institute of Microbiology, Chinese Academy of Sciences, Beijing, People’s Republic of China

**Keywords:** *Akkermansia muciniphila*, Lipooligosaccharide, Atherosclerosis, IL-22, Gut microbiota

## Abstract

As an important and beneficial gut commensal, *Akkermansia muciniphila* plays a crucial role in regulating host metabolism and immunity. Lipooligosaccharides from *A. muciniphila* (ALOS) show anti-obesity effects in high-fat diet-fed mice. Herein, we investigated the chemical characteristics of core oligosaccharides of ALOS and explored its anti-atherosclerotic efficacy. The LC-Q-TOF-MS analysis indicated a high structural diversity of core oligosaccharides in ALOS, comprising fourteen distinct oligosaccharide species with different degrees of phosphorylation. Functionally, administration of ALOS significantly attenuated hyperlipidemia and reduced atherosclerotic plaque burden in high-fat diet-fed *ApoE*^*−/−*^ mice. The improvement of these metabolic symptoms was related to the restoration of intestinal barrier integrity. Mechanistically, ALOS upregulated the IL-23/IL-22 immune axis, which in turn promoted intestinal epithelial repair and modulates the microbiota. ALOS intervention reshaped the gut microbiota composition by enriching beneficial genera such as *Bifidobacterium longum*, *Roseburia intestinalis*, and *Oscillibacter sp.*, while suppressing potential pathobionts. Our findings highlight the structural diversity and anti-atherosclerotic effect of lipooligosaccharides from *A. muciniphila*.

**Graphical Abstract:**

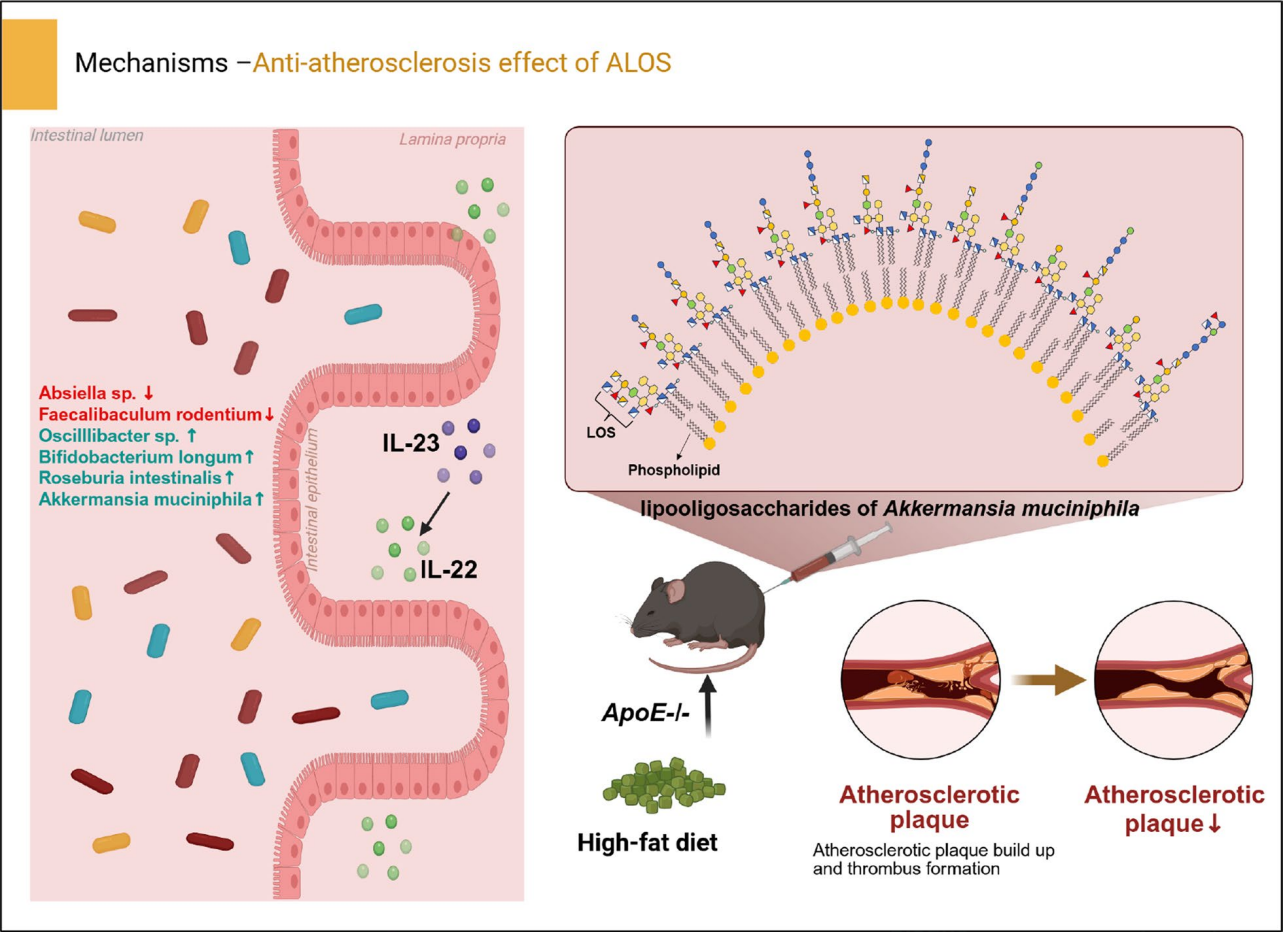

**Supplementary Information:**

The online version contains supplementary material available at 10.1007/s13659-026-00612-4.

## Introduction

Lipopolysaccharide (LPS), a conserved microbial component, plays a pivotal role in training and shaping mucosal immunity through its interaction with pattern recognition receptors (e.g., TLR-4 and TLR-2), a process critical for gut immune homeostasis. LPS molecules have a common chemical architecture, composed of a lipid A moiety and a saccharide chain[1, 2]. Two groups of LPS have been identified in Gram-negative bacteria, including the smooth-type LPS (S-LPS) possessing a repeating O-polysaccharide domain (O-chain) in the saccharide chain and the rough-type LPS (R-LPS) without O-chain. R-LPS are also termed as lipooligosaccharides (LOS). Gut commensal bacteria-derived LOS from *Bacteroides dorei, Bacteroides vulgatus*, and *Parabacteroides goldsteinii* have been chemically characterized and demonstrated to have beneficial effects on the host, enhancing immunological tolerance and alleviating chronic obstructive pulmonary disease [3–5]. The previous work on *Segatella copri* LPS revealed that a hypo-acylated and hypo-phosphorylated lipid A, coupled with a mannose- and glucose-rich oligosaccharide core, weakly activated the TLR4 receptor and promotes a moderate immune response [6]. Similarly, Cho et al. demonstrated that tetra-acylated monophosphorylated lipid A (4A-MPLA) from *Bacteroides fragilis* induced a sustained IFN-β response and fosters the expansion of colonic RORγt + Tregs, thereby suppressing inflammation [7]. Hypoacylated derived LPS from *Alcaligenes* enhanced the IgA production without excessive inflammatory responses via weak TLR4 agonist activity [8].

*Akkermansia muciniphila* is an important gut mucous layer-colonized bacterium of Verrucobacteria, rich in the gut microbiota of healthy humans. It has been proposed as the representative of the next generation of probiotics for control of obesity[9, 10]. Early studies have demonstrated that oral supplementation of this bacterium improves insulin resistance in both animal and human clinical trials and restores mucosal integrity[11, 12]. Several bioactive components, including the outer pili-like protein Amuc_1100, the branched diacyl phosphatidylethanolamine, tripeptide, and the threonyl-tRNA synthetase, have been reported from *A. muciniphila*[13–16].

In our early work, a hypoacetylated and *mono/bis*-phospharylated LOS was identified from *A. muciniphila* (ALOS) [17]. ALOS is a weak TLR-4 agonist that reduces metabolic disorders in obese mice via activating the TLR-4-IL-23-IL-22 signaling pathway. In this work, we further revealed the structural diversity of oligosaccharide moieties in the ALOS by MS/MS analysis. In addition, we evaluated the anti-atherosclerotic effect of ALOS, which demonstrated protective effects by reducing hyperlipidemia and attenuating atherosclerotic injuries.

## Results and discussions

### MS/MS analysis of oligosaccharides in the LOS of *A. muciniphila*

**Fig. 1:**
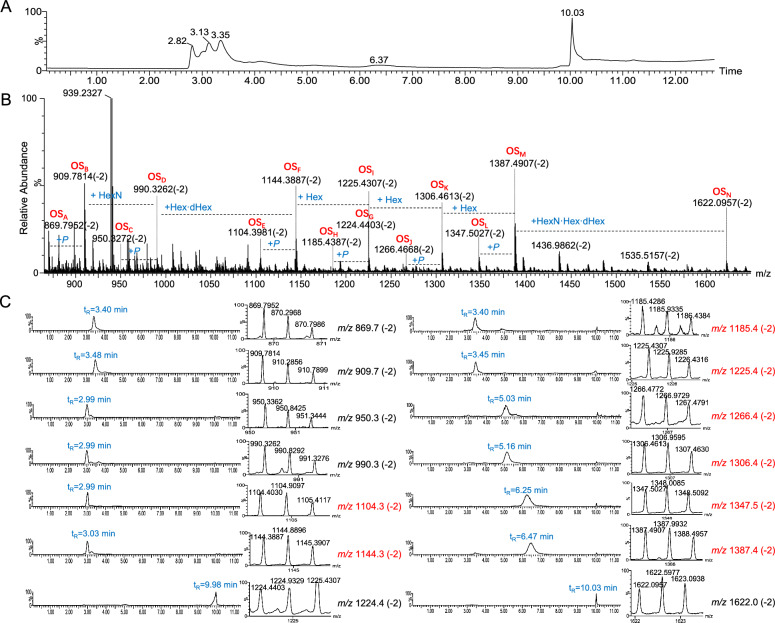
Analysis of core oligosaccharide in the LOS from *A. muciniphila* by LC-Q/TOF–MS in the negative ion mode. **A** The total ion chromatogram (TIC) recorded for the core oligosaccharide from *A. muciniphila*. **B** Double-charged molecular ion peaks detected in the LC-Q/TOF-MS spectrum. **C** The extracted ion chromatograms (EICs) of different core oligosaccharide species from *A. muciniphila*.

**Fig. 2:**
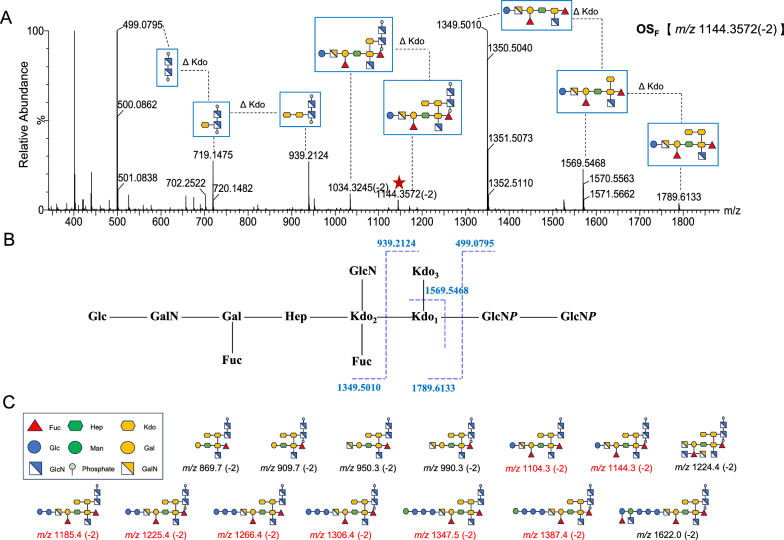
The CID MS/MS spectrum of the oligosaccharide OS_F_ and proposed structures of oligosaccharides detected in *A. muciniphila. A* CID MS/MS spectrum from the double-charged mother ion at *m/z* 1144.3. **B** A proposed fragmentation pattern for OS_F_. **C** The proposed structural sequences of the core oligosaccharide from *A. muciniphila* sketched according to the symbology of monosaccharide residues.“Hex”, hexose; “Hep”, heptose; “Fuc”, fucose; “HexN”, hexosamine; “*P*”, phosphate; “Kdo”, 3-deoxy-D-manno-2-oct-ulopyranosonic acid, respectively.

**Scheme1.**
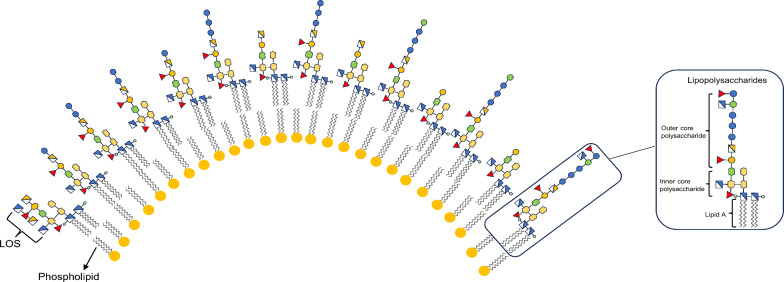
Proposed distribution of lipooligosaccharides on the outer membrane of *A. muciniphila*

In early reports, oligosaccharide moieties in the ALOS were found to be diverse, with two major oligosaccharides OS_G_ and OS_N**,**_ determined by NMR and MS analysis [17, 18]. In the current work, to clearly explore the structural diversity of ALOS*,* the fully deacylated lipopolysaccharides from *A. muciniphila* were characterized using LC-Q-TOF mass spectrometry. Mass spectra in negative-ion modes and collision-induced dissociation (CID) fragmentation experiments were optimized for each selected parent ion that provided extensive information to elucidate sequences of oligosaccharide chains (**OS**). As shown in the ion chromatogram, the oligosaccharides showed a high heterogeneity resulting from the presence of fourteen distinct OS species at *m/z* 869.7, *m/z* 909.7, *m/z* 950.3, *m/z* 990.3, *m/z* 1104.3, *m/z* 1144.3, *m/z* 1185.3, *m/z* 1225.3, *m/z* 1224.4, *m/z* 1266.4, *m/z* 1306.4, *m/z* 1347.5, *m/z* 1387.4 and *m/z* 1622.0 (Fig. 1A–C). Six of these variants (*m/z* 869.7, *m/z* 909.7, *m/z* 950.3, *m/z* 990.3, *m/z* 1224.4, and *m/z* 1622.0) were also observed in our previous report based on in-source fragmentation HPLC–ESI–MS analysis [14]. Based on the doubly-charged ions detected in the mass range of *m/z* 850–1650, the reported monosaccharide composition in ALOS, and the structure of OS_G_ and OS_N_ [17, 18]_**,**_ the sugar sequences in OS_A_-OS_N_ was elucidated. For example, the oligosaccharide OS_F_ was detected at *m/z* 1144.3572 [M-2H]^2−^ with a retention time of t_R_ = 3.03 min, which was deduced to be the dodecasaccharide composed of three 3-deoxy-D-*manno*−2-oct-ulopyranosonic acid moieties (Kdo-1, 2, and 3), two fucoses, one heptose, four hexosamines, two hexoses, and two phosphoric acid moieties (Supplementary Table 1). The CID MS/MS spectrum of **OS**_**F**_ indicated the presence of daughter ions at *m/z* 1034.3 (−2), 1789.6, 1569.5, 1349.5, 939.2, 719.1, and 499.0, as shown in Fig. 2A, B. The occurrence of a fragment ion at *m/z* 1034.3 (−2) was due to the loss of one Kdo moiety from the double-charged parent ion at *m/z* 1144.3572 (−2). Two paired fragment ions (*m/z* 1789.6 and 499.0; 1569.5 and 939.2) were formed due to the cleavage of the glycosidic bond between GlcN*P* and Kdo-1, between Kdo-1 and Kdo-2 from **OS**_**F**_, respectively. The ions at *m/z* 1569.5 and 719.1 were derived from the daughter ions at *m/z* 1789.6 and 939.2 due to loss of one Kdo, respectively. By the similar way, the structural sequences of oligosaccharides **OS**_**E**_ and **OS**_**H-M**_ corresponding to *m/z* 1104.3 (−2), 1185.3 (−2), 1225.3 (−2), 1266.4 (−2), 1306.4 (−2), 1347.5 (−2), and 1387.4 (−2) were determined (Fig. 2C, Supplementary Fig. 1A-M, and Supplementary Table 1). Of note, the inner core oligosaccharide of ALOS was found to be composed of three Kdo, one fucose, one heptose, and one hexosamine. The structures for oligosaccharides (OS_A_-OS_F_ and OS_H_-OS_M_) need to be further assigned by sophisticated purification and NMR data elucidation. The diverse oligosaccharides reflect the structural diversity of LOS in the commensal *A. muciniphila* (Scheme 1), which is deduced to play essential roles in the regulation of bacterial colonization and immune regulation.

### ALOS inhibits atherosclerosis in ***ApoE***^***−/−***^ mice

**Fig. 3:**
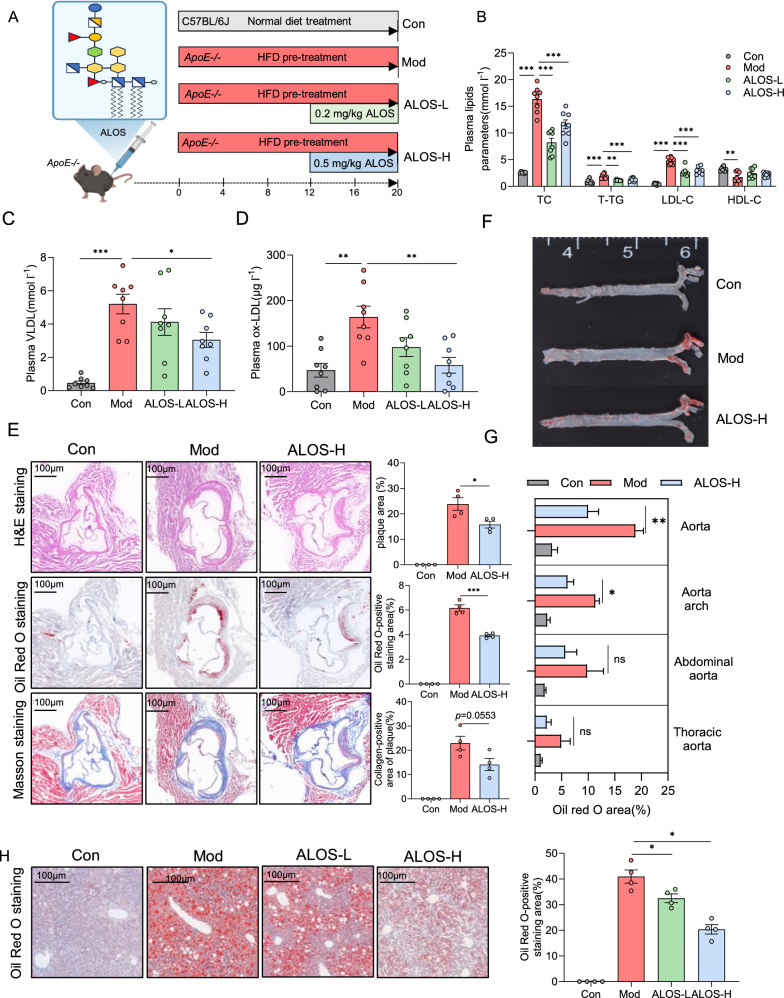
ALOS attenuates atherosclerosis in *ApoE*^*−/−*^ mice. **A** Experimental design showing groups and durations (n = 8). ALOS: lipooligosaccharides derived from *A.muciniphila*. ALOS-L: 0.2 mg/kg ALOS treated- *ApoE*^*−/−*^. ALOS-H: 0.5 mg/kg ALOS treated- *ApoE*^*−/−*^. **B** Plasma lipid parameters (TC, T-TG, LDL-C, and HDL-C). **C** Plasma very low-density lipoprotein (VLDL). **D** Oxidized low-density lipoprotein (ox-LDL). **E** Representative H&E staining, Oil Red O staining, and Masson staining of cross-sections of aortic roots and quantitative data of plaque area, ecrotic core area, and collagen-positive area (scale bar, 100 μm, n = 4). **F** Lipid content of the whole aorta visualized by staining with Oil Red O. **G** The quantitative data of Oil Red O-positive staining area; n = 4 mice for each group. **H** Representative Oil Red O staining of liver (scale bar, 100 μm, n = 4). *P < 0.05; **P < 0.01. One-way ANOVA (with Tukey's HSD) test in B–H.

In previous studies, *A. muciniphila* was reported to attenuate atherosclerotic lesions by reducing metabolic endotoxemia-induced inflammation through restoration of the gut barrier[19]. Interleukin (IL)−23 and its downstream target IL-22 have been reported to limit the development of atherosclerosis by inhibiting the pro-atherosclerotic microbiota[20]. Based on the beneficial effect of *A. muciniphila* on atherosclerosis and the immunological trait of ALOS, we explored the possible role of ALOS in atherosclerosis. *ApoE*^*−/−*^ mice fed with the high-fat diet were intraperitoneally treated with ALOS or vehicle (PBS) for a period of 8 weeks (Fig. 3A). By the end of treatment, plasma lipid levels were significantly improved by ALOS. In comparison with vehicle-treated *ApoE*^*−/−*^ mice, reductions in the levels of total cholesterol (TC; 55% and 29%), low-density lipoprotein cholesterol (LDL-C; 44% and 38.9%), total triglycerides (T-TG; 40% and 32%) were achieved by ALOS at doses of 0.2 and 0.5 mg kg^−1^, respectively (Fig. 3B). The levels of very-low-density lipoprotein (VLDL) and oxidized LDL (ox-LDL) were also significantly reduced by ALOS in a concentration-dependent manner (Fig. 3C–D). The high-fat diet induced formation of atherosclerotic lesions, as demonstrated by hematoxylin and eosin staining, Oil Red O staining, and Masson staining of aortic root regions (Fig. 3E). Compared to Mod, treatment with ALOS substantially reduced the aortic root lesion (Fig. 3E). After 12 weeks of high-fat diet, the accumulation of lipid in the whole aorta was shown in Fig. 3F. Treatment with ALOS substantially reduced the lesion area, necrotic core burden, and the content of collagen (Fig. 3E–G). In addition, ALOS also reduced the lipid accumulation in the liver (Fig. 3H). Based on these results, ALOS showed promising anti-atherosclerosis efficacy.

### ALOS ameliorates gut barrier function and promotes IL-22 production

**Fig. 4:**
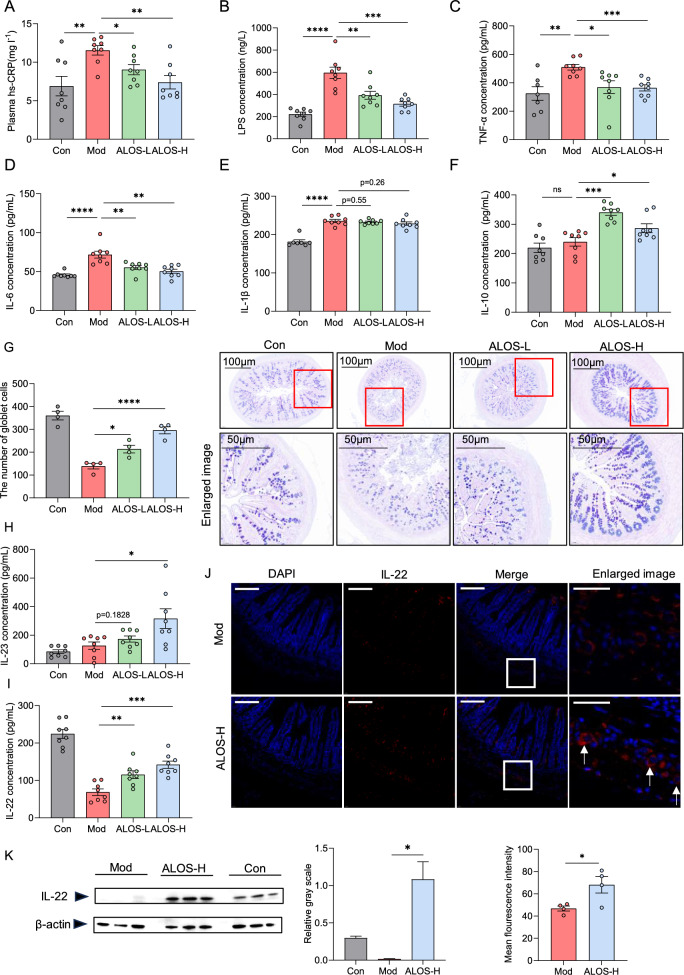
ALOS ameliorates gut barrier function and promotes IL-22 production. **A** C-reactive protein (hs-CRP). **B** LPS. **C** Tumour necrosis factor-alpha (TNF-α). **D** Interleukin-6 (IL-6). **E** Interleukin 1β (IL-1β). **F** Interleukin 10 (IL-10). **G** Representative AB-PAS staining of ileum pathological sections (scale bar, 100 μm, n = 4). **H** Plasma IL-23. **I** Plasma IL-22. **J** Representative immunofluorescent staining of IL-22 in the ileum. n = 4 mice for each group. Scale bar, 20 μm. **K** Western blotting of the IL-22 and β-actin levels in the ileum tissue of *ApoE*^*−/−*^ mice. *P < 0.05; **P < 0.01. One-way ANOVA (with Tukey's HSD) test in A–I. Unpaired t-test in J–K.

Endotoxemia caused by the increased intestinal epithelial permeability due to long-term high-fat diet is recognized as one of the pathogenesis mechanisms underlying the development of atherosclerosis[21, 22]. Compared with atherosclerosis model mice, ALOS-treated mice showed significantly lower plasma LPS levels and high-sensitivity C-reactive protein (hs-CRP) (Fig. 4A–B). Furthermore, ALOS decreased the plasma levels of proatherogenic cytokines, such as IL-6, TNF-α, and increased the levels of anti-inflammatory IL-10 (Fig. 4C–D, and F), while the level of IL-1β remained unaltered (Fig. 4E). The intestinal epithelium serves as the first defense barrier of the gastrointestinal tract and its compromise directly induces inflammation. AB-PAS staining revealed significant reductions of intestinal tissue in goblet cell count in the atherosclerosis model group (Fig. 4G), while ALOS supplementation mitigated the damage to epithelial integrity. In our earlier study on metabolic syndrome, it has been confirmed that ALOS has a weak TLR4 agonist and can elicit the IL-23/IL-22 axis, which is essential for maintaining the integrity of the intestinal barrier and modulation of gut microbiota[17]. We hypothesize that IL-23/IL-22 might be one of the mechanism by which ALOS suppress atherosclerosis. In *ApoE*^*−/−*^ mice, the plasma level of IL-22 showed a marked decrease compared to that of normal mice. Compared to atherosclerosis model mice, a high dose of ALOS treatment significantly increased the plasma levels of IL-23 and IL-22 (Fig. 4H–I). The production of intestinal IL-22 was also reduced in the mice with atherosclerosis, while ALOS treatment significantly increased the protein levels of IL-22, as shown by immunofluorescent staining and WB (Fig. 4J–K). These results suggest that the repair of ALOS on the intestinal barrier in atherosclerotic mice is mechanistically related to the activation of the IL-23/IL-22 pathway.

### ALOS reshapes the structure of the intestinal microbiota

**Fig. 5:**
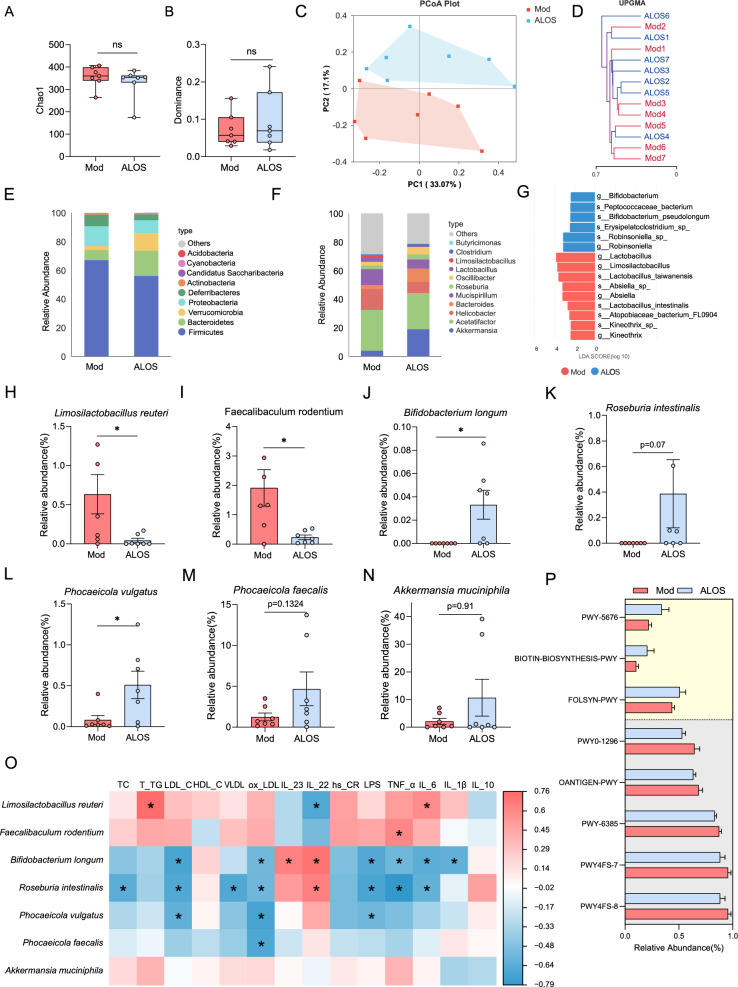
ALOS reshapes the structure of the intestinal microbiota. **A** The Chao 1 index. **B** The dominance index. **C** Principal coordinate analysis. **D** UniFrac distance-based unweighted pair-group method with arithmetic (UPGMA) means analysis. The shorter the branch length between samples, the higher the similarity between the two samples. **E** Relative abundance of gut microbiota at the phylum level. **F** Relative abundance of gut microbiota at the generic level. **G** LEfSe analysis of microbiota. **H–N** The relative abundance of differentially abundant flora identified at different taxonomic levels. **O** Spearman’s correlation analyses with serum biochemical parameters and IL‐22 levels. **P** Predict microbial function on the METACYC database. PWY-5676 (acetyl-CoA fermentation to butanoate II), BIOTIN-BIOSYNTHESIS-PWY (biotin biosynthesis I), FOLSYN-PWY (superpathway of tetrahydrofolate biosynthesis and salvage), PWY0-1296 (purine ribonucleosides degradation), OANTIGEN-PWY (O-antigen building blocks biosynthesis (*E. coli*)), PWY-6385 (peptidoglycan biosynthesis III (mycobacteria)), PWY4FS-7 (phosphatidylglycerol biosynthesis I (plastidic)), PWY4FS-8 (phosphatidylglycerol biosynthesis II (non-plastidic)). *P < 0.05; **P < 0.01. Unpaired t-test in A–B, H–J, L–O. Mann–Whitney U test in K, P.

Given the role of intestinal IL-22 in regulating antimicrobial peptides production and shaping gut microbiota composition[23–26], we next examined the influence of ALOS on the composition of intestinal microbiota in *ApoE*^*−/−*^ mice by the high-throughput sequencing of 16S rRNA in the cecal content. A total of 1256267 sequences were obtained after denoising with DADA2, averaging 89,733 sequences in each sample. Treatment with ALOS did not affect the α-diversity of the bacterial community, as indicated by the Chao1 index and Dominance index, suggesting that ALOS did not change the richness and diversity of the gut microbiome (Fig. 5A–B). Principal coordinates analysis (PCoA) analysis revealed a distinct clustering of gut microbiota composition between the two groups (Fig. 5C), and the UPGMA clustering analysis also showed a clear separation between the model and ALOS groups (Fig. 5D). To further investigate specific alterations of bacterial communities, we compared the relative abundance of the major microbial taxa at the phylum, genus, and species levels. Firmicutes, Bacteroidetes, and Verrucomicrobia were the dominant bacterial phyla in both groups (Fig. 5E). The Firmicutes to Bacteroidetes (F/B) ratio was significantly lower in the ALOS-treated groups (3.18) compared to the mod groups (9.53), corresponding to a 66.63% decrease (Fig. 5E). Meanwhile, ALOS mainly up-regulated the abundance of the genus of *Akkermansia*, *Roseburia*, *Oscillibacter*, and *Butyricimonas* and decreased the abundance of pathogenic bacteria belonging to *Helicobacter* and *Mucispirillum* (Fig. 5F). The linear discriminant analysis effect size (LEfSe) identified 15 operational taxonomic units (OTUs) altered by ALOS, with a significant increase in *Bifidobacterium*, *Robinsoniella*, and a decrease in *Absiella* (Fig. 5G). As to the abundance of species, ALOS treatment decreased the relative abundance of *Limosilactobacillus reuteri*, and *Faecalibaculum rodentium*, while enriching the abundance of *Bifidobacterium longum*, *Roseburia intestinalis*, *Phocaeicola vulgatus*, *Phocaeicola faecalis*, and *Akkermansia muciniphila* (Fig. 5H–N). These shifts conferred metabolically benefits. *F. rodentium*, which was decreased by ALOS, contributes to the susceptibility of metabolic disorders by suppressing segmented filamentous bacteria and reducing intestinal Th17 cells [27]. Conversely, *B. longum* enriched by ALOS exhibited beneficial effects against metabolic disorders by regulating the mRNA expression of renin-angiotensin system factors and ameliorating lipid and glucose levels [28]. *R. intestinalis*, a promising next-generation probiotic, reinforced barrier function and suppressed pro-inflammatory signaling via butyrate production[29].

To further investigate the correlation of the key bacteria induced by ALOS and the atherosclerosis-associated symptoms, a Spearman correlation analysis was conducted. As a result, *L. reuteri*, *Absiella sp.*, and *F. rodentium*, which were decreased by ALOS, were positively correlated with the parameters of atherosclerosis, while the ALOS-enriched bacteria, such as *Oscilllibacter sp.*, *B. longum*, *R. intestinalis*, *P. vulgatus*, and *P. faecalis*, were negatively correlated with the symptoms of atherosclerosis (Fig. 5O). To predict the functional alteration in the gut microbiota after treatment, the PICRUSt2 analysis was employed. The results revealed that ALOS upregulated the metabolic pathways of PWY-5676 (acetyl-CoA fermentation to butanoate II), BIOTIN-BIOSYNTHESIS-PWY (biotin biosynthesis I) and FOLSYN-PWY (superpathway of tetrahydrofolate biosynthesis and salvage), which could benefit for ameliorating atherosclerosis [30–32], while downregulated the pathways, such as PWY0-1296 (purine ribonucleosides degradation), OANTIGEN-PWY (O-antigen building blocks biosynthesis (*E. coli*)), PWY-6385 (peptidoglycan biosynthesis III (mycobacteria)), PWY4FS-7(phosphatidylglycerol biosynthesis I (plastidic)), PWY4FS-8(phosphatidylglycerol biosynthesis II (non-plastidic)) (Fig. 5P). Collectively, these data suggest that ALOS treatment partially regulated the structure and functions of gut microbiota in *ApoE*^*−/−*^ mice. In conclusion, ALOS treatment ameliorates the dysbiosis of gut microbiota in mice with atherosclerosis, thereby contributing to the alleviation of atherosclerotic injuries.

## Discussions

In this study, the distribution and diversity of oligosaccharide chains in the lipooligosaccharids of *A. muciniphila* was proposed. MS/MS analysis of oligosaccharides in the ALOS identified fourteen *mono/bis*-phosphorylated oligosaccharide species. Based on our prior and current findings demonstrating that ALOS mediated activation of the IL-23/IL-22 in both obesity and *ApoE*^*−/−*^ mice models [17, 18], we propose that the unique structural features and high diversity of ALOS may coordinately contribute to its ability to elicit a balanced immune activation, favoring the IL-23/IL-22 pathway over pro-inflammatory cytokine cascades. Consistent with their unique chemical structures and activation of IL-23/IL-22 immune response, the lipooligosaccharides derived from *A. muciniphila* are associated with attenuated hyperlipidemia, reduced plaque burden, improved intestinal barrier markers, and a reshaped gut microbiota in *ApoE*^*−/−*^ mice.

As a relatively weak TLR4 agonist, ALOS exhibits favorable safety profiles, as demonstrated in our previous studies through acute endotoxin toxicity models, comparing to the conventional LPS from *Escherichia coli* [17]. LPS molecules are easily degraded in the acidic gastric environment, limiting oral bioavailability. Consequently, intraperitoneal administration was used in the current study. The well-established crosstalk between immunity and the gut microbiota[33] suggests that systemic ALOS-induced activation of the IL-23/IL-22 axis may promote the expansion of metabolically beneficial gut commensals, such as *Bifidobacterium longum*, *Roseburia intestinalis*, *Phocaeicola vulgatus*, *Phocaeicola faecalis*, and *A. muciniphila.*

Despite these promising findings, several limitations of the current study should be acknowledged. The decomposition of LPS under acidic condition hinders the application of oral delivery, a preferable route for gut microbiota modulation and relatively safe for therapeutic applications. In the following study, coating ALOS with suitable material in formulation to avoid acidic condition in stomach is necessary for establishing an oral delivery system for ALOS. In early studies, we and other researchers have chemically identified two main oligosaccharides in LOS of *A. muciniphila* through detailed analysis of MS and NMR data[17, 18]. However, duo to its high heterogeneity, separation of other oligosaccharides is unsuccessful after great efforts. In this work, we turned to LC–MS/MS analyses to show oligosaccharide diversity in ALOS. The obtained MS/MS data together with previously reported structural information for two main oligosaccharides (OS_G_ and OS_N_) revealed structural diversity in oligosaccharides of ALOS, but the exact linking sites between sugar units and complete NMR assignment remain to be confirmed. To achieve purification of these unique oligosaccharides(OS_A_-OS_F_ and OS_H_-OS_M_), new sophisticated separation approaches are needed to be developed. Moreover, further investigations are required to establish the safety, immune activation thresholds, and the translational dosingof ALOS.

In conclusion, our study revealed the structural characteristics and diversity of oligosaccharides in the lipooligosaccharides of *A. muciniphila*, demonstrated the anti-atherosclerotic activity and gut microbiota modulation of ALOS in mice. These findings advance our understanding of microbiome-host crosstalk in cardiovascular disease and suggest the therapeutic potential of gut bacterial glycolipids.

## Experimental sections

### Isolation and characterization of oligosaccharides from LOS of *A. muciniphila*

*A. muciniphila* HW07 strain was cultured anaerobically at 37 °C in modified brain–heart infusion (BHI) broth. The extraction and purification of LOS from *A. muciniphila* (ALOS) were conducted according to our early study [17]. The fully deacylated oligosaccharides linked to the lipid A sugar skeleton were subsequently obtained from the purified ALOS by reduction and alkaline hydrolysis procedure. Briefly, the purified ALOS (1 mg) was treated with hydrazine solution and stirred (37 ℃, 1.5 h). Subsequently, the resulting reduced product was then heated with 4 M KOH (120 ℃, 16 h). After cooling, the mixture was allowed to precipitate. The aqueous phase containing the deacylated oligosaccharide was collected, neutralized with 2 M HCl, and dialyzed (molecular mass cut-off: 500 Da) to remove salts and small molecules. The Q-TOF MS and MS/MS analysis of the deacylated oligosaccharides was acquired on a Water Xevo G3 Q-TOF mass spectrometry in the negative ion mode. For CID-MS/MS analysis, the sample was dissolved in water and analyzed using a Shim-pack GIST C18-AQ column (250 × 4.6 mm; 5 μm; SHIMADZU, Japan) at a flow rate of 0.3 mL/min. The mass spectrometer was set to scan over an m/z range of 60–3000. A gradient elution beginning with Solvent A (0.01% formic acid) for 3 min, then with an increase of Solvent B (acetonitrile) to 100% (v/v) over 10 min, followed by 100% B for 5 min and a decrease to 100% A in 8 min, was applied.

### Mice

C57BL/6 J and *ApoE*^*−/−*^ mice (6–8 weeks, male) were obtained from the Experimental Animal Center, Chinese Academy of Medical Sciences. All animals were normally housed under specific pathogen-free (SPF) conditions. The chow diet (13.5% calories from fat) and a high-fat diet (HFD, 60% kcal fat, D12492, New Brunswick) were used for feeding. Experiments were conducted according to approval by the Institute of Microbiology, Chinese Academy of Sciences (IMCAS).

### The effects of ALOS on high-fat diet-induced *ApoE*^*−/−*^ mice

C57BL/6 J fed with a normal diet (ND), *ApoE*^*−/−*^ mice fed with HFD at 8 weeks of age, diet pretreatment for 12 weeks, and then divided into three groups. Intraperitoneal injection of ALOS 0.2 mg/kg (ALOS-L) or 0.5 mg/kg (ALOS-H) once every two days for 8 weeks.

### Biochemical analyses

The interleukin-6 (IL-6) (SEKM-0007; Solarbio), tumor necrosis factor-alpha (TNF-α) (RK00027; Abclonal), interleukin-1β (IL-1β) (MM-0040M1; MEIMIAN), interleukin-10 (IL-10) (1418033–25-6; Solarbio), interleukin-22 (IL-22) (E-MSEL-M0040; Elabscience BiotechnologyCo., Ltd.), interleukin-23 (IL-23) (RK00017; Abclonal), oxidized LDL (ox-LDL), very low density lipoprotein cholesterol (VLDL) (ml037709-2; Mlbio), and lipopolysaccharide (LPS) (WLCSJZF202535; AMOY LUNCHANGSHUO) levels in the plasma and ileum were determined in commercial ELISA kits. High-sensitivity C-reactive protein (Hs-CRP) was determined by commercial ELISA kits (EnBo, Beijing, China). The total cholesterol (T-CHO), triglycerides (TG), low-density lipoprotein cholesterol (LDL-C), and free fatty acid (FFA) levels in plasma were determined by commercially available kits (Nanjing Jiancheng Bioengineering Institute, Jiangsu, China).

### Histological analysis

The aortic roots, liver, adipose tissues, and intestinal tissues were isolated and fixed in 4% paraformaldehyde solution. The aortic roots were stained with H&E, Oil Red O, or Masson’s; the livers were stained with Oil Red O; the intestinal tissues were stained with AB-PAS. The lesion area, the number of goblet cells, the relative area of Oil Red O, and Collagen volume fraction (CVF) were measured using ImageJ software.

### DNA extraction and 16S rRNA sequencing analysis

According to the manufacturer’s instructions, fecal DNA was extracted using the QIAamp Fast DNA stool Mini Kit (Qiagen, Cat# 51604). Just as in previous study[34], Primers for the 16S rRNA variable region 3–4 (V3-V4) were 338 F (5′-ACTCCTACGGGAGGCAGCA-3′) and 806R (5′-GGACTACHVGGGTWTCTAAT-3′). DNA library was performed on an Illumina NovaSeq sequencing platform.

### Immunofluorescence staining analysis

The fresh intestinal tissues were isolated and immediately fixed in 4% paraformaldehyde solution for 24 h and blocked with 5% bovine serum albumin for 30 min. Then, the 3–5 μm thick slides were incubated at 4 °C overnight with primary antibodies: anti-IL-22 rabbit pAb (R30198; Zenbio), followed by the secondary antibody for 50 min at room temperature with goat anti-rabbit IgG-Cy3 (Servicebio, GB21303). The ImageJ software was used to quantify the images.

### Western blotting

After the ileal tissue of mice was homogenized in the cold RIPA lysis buffer at 50 mg/L, the protein concentration of the supernatant was determined by the BCA method. The protein was isolated by sodium dodecyl sulfate polyacrylamide gel electrophoresis (SDS-PAGE) (36276ES10; Yeasen) and transferred to polyvinylidene fluoride (PVDF, Servicebio; G6044-0.45) membrane. First, the membranes were closed with 5% non-fat milk at room temperature for 1 h. Secondly, the membranes were incubated overnight at 4 ℃ with different primary antibodies: anti-IL-22 (R30198; Zenbio) and β-actin (700068; Zenbio). Finally, HRP-linked goat anti-rabbit IgG (511203; Zenbio) secondary antibody was incubated for 1 h. Protein was visualized using ECL Plus Western Blot assay (36208ES7636208ES76; Yeasen). β-actin was used to normalize the expression of IL-22.

### Statistical analysis

Correlations between species and factors were analyzed using Spearman's correlation coefficient. Spearman's correlation analysis calculates Spearman's ρ, ρ = 1 indicates a completely positive linear correlation, ρ = −1 indicates a complete negative linear correlation, and ρ = 0 indicates no linear correlation. The normality of the distribution was tested by the Kolmogorov–Smirnov test. Comparisons between the two groups are represented by Welch's t‐test, unpaired t‐test, and Mann–Whitney U test. Welch's t‐test required normality in both groups but did not require equal variances. The unpaired t‐test required both normality and equal variances. The Mann–Whitney U test required neither normality nor equal variances. One‐way ANOVA test (post‐event verification method: Tukey's HSD) was used for multiple comparisons. The data in the figure were expressed as mean ± SEM. GraphPad Prism 9.0 and ImageJ were used to analyze all the data and showed as: ns *p* > 0.05, *p < 0.05, **p < 0.01, ***p < 0.001, ****p < 0.0001.

## Supplementary Information

Supplementary Material 1

## Data Availability

Raw 16S rDNA data had been deposited on NCBI at https://submit.ncbi.nlm.nih.gov/subs/bioproject/SUB15789623/overview with BioProject submission: SUB15789623. All other data supporting the findings of the study are available within the article and its supplementary information files, or from the corresponding authors upon reasonable request.
