# Supplementary material for: Diversity of oligosaccharides in lipooligosaccharides of Akkermansia muciniphila and its anti-atherosclerotic activity
Source: Nat Prod Bioprospect. 2026 May 2;16(1):58. doi: 10.1007/s13659-026-00612-4 (PMC13135019; doi:10.1007/s13659-026-00612-4)
Supplement: Supplementary file 1 — Supplementary Material 1 [file 13659_2026_612_MOESM1_ESM.docx]

**Supplemental Information**

**Diversity of oligosaccharides in lipooligosaccharides of *Akkermansia muciniphila* and its anti-atherosclerotic activity**

Yuting Zhang^1,2,3#^, Wang Dong^1,2,3#^, Jinghan Lin^1,2,3#^, Jingzu Sun^1,3^, Ruopeng Yin^1,2,3^, Xun Lv^1,3^, Wenzhao Wang^1^, Tao Wang^1,3*^ and Hongwei Liu^1,2,3*^

**^#^**Yuting Zhang, Wang Dong, and Jinghan Lin have contributed equally to this study.

Correspondence:

Tao Wang

wangtao@im.ac.cn

Hongwei Liu

liuhw@im.ac.cn

The full list of author information is available at the end of the article.

**Supplementary Figures**

**Supplementary Figure 1. Negative-ion mode collision-induced dissociation (CID) MS/MS spectra** **and the proposed sugar sequence of oligosaccharides.** The glycan chains of OS from *A. muciniphila* are presented as the symbology of mono-sugar units. The double-charged ion at *m/z* 869.7 (A, OS_A_), 909.7 (B, OS_B_), 950.3 (C, OS_C_), 990.3 (D, OS_D_), 1104.3 (E, OS_E_), 1224.4 (F, OS_G_), 1185.3 (G, OS_H_), 1225.3 (H, OS_I_), 1266.4 (I, OS_J_), 1306.4 (J, OS_K_), 1347.4 (K, OS_L_), 1387.4 (L, OS_M_), and 1622.0 (M, OS_N_) were detected, respectively.

**Supplementary Tables**

**Supplementary Table 1 The proposed interpretation of the core oligosaccharide species in the LOS from *A. muciniphila***
